# JNK2 downregulation promotes tumorigenesis and chemoresistance by decreasing p53 stability in bladder cancer

**DOI:** 10.18632/oncotarget.9046

**Published:** 2016-04-27

**Authors:** Chun-Wu Pan, Hailong Liu, Yu Zhao, Chenchen Qian, Liguo Wang, Jun Qi

**Affiliations:** ^1^ Department of Urology, Xinhua Hospital, Shanghai Jiao Tong University School of Medicine, Huangpu, Shanghai 200092, China; ^2^ Department of Biochemistry and Molecular Biology, Mayo Clinic College of Medicine, Rochester, MN 55905, US; ^3^ Department of Gastroenterology, The First Affiliated Hospital of Sun Yat-Sen University, Guangzhou, Guangdong 510080, China; ^4^ Division of Biomedical Statistics and Informatics, Mayo Clinic College of Medicine, Rochester, MN 55905, US

**Keywords:** JNK2, p53, bladder cancer, tumorigenesis, chemoresistance

## Abstract

Bladder cancer is one of the most common malignancies of the urinary system, and the 5-year survival rate remains low. A comprehensive understanding of the carcinogenesis and progression of bladder cancer is urgently needed to advance treatment. c-Jun N-terminal kinase-2 (JNK2) exhibits both tumor promoter and tumor suppressor actions, depending on tumor type. Here, we analyzed the JNK2 function in bladder cancer. Using gene expression microarrays, we demonstrated that JNK2 mRNA is downregulated in an orthotopic rat model of bladder cancer. JNK2 protein levels were lower in rat and human bladder cancer tissues than in normal tissues, and the levels correlated with those of p53. Moreover, JNK2 phosphorylated p53 at Thr-81, thus protecting p53 from MDM2-induced proteasome degradation. Decreased expression of JNK2 in T24 cells conferred resistance to cell death induced by mitomycin C. Furthermore, lower JNK2 expression was associated with poorer overall survival among patients who underwent radical cystectomy. These results indicate that JNK2 acts as a tumor suppressor in bladder cancer, and that decreased JNK2 expression promotes bladder cancer tumorigenesis.

## INTRODUCTION

Bladder cancer is one of the most common malignant tumors of the urinary system [1]. Non-muscle invasive bladder cancer (NMIBC) accounts for nearly 70% of these cases, which can be easily resected through transurethral resection of bladder tumor (TURBT), but recur frequently (50-70%). Ten percent to 5% of NMIBCs progress to muscle invasive bladder cancer (MIBC), which has a 5-year survival rate of less than 50%, even if treated with radical cystectomy (RC) combined chemotherapy [2]. Bladder cancer treatment has not improved for decades, and new approaches are needed [3]. A comprehensive understanding of the carcinogenesis and progression of bladder cancer is urgently needed to advance treatment.

The c-Jun N-terminal kinase (JNK) family, a subfamily of the MAPKs, participates in a variety of cell responses, including proliferation, differentiation, and cell death and survival. As a result, abnormal JNK function leads to diverse diseases, including cancer [4]. The JNK family contains three proteins, of which JNK1 and JNK2 are widely expressed in most tissues, while JNK3 is found only in brain, heart and testes [5]. The role of JNK1 and JNK2 in cancer is complicated and controversial. JNK1 is thought to function as a tumor promoter in lung cancer and primary hepatocellular carcinoma [6, 7], and JNK2 promotes carcinogenesis in skin cancer and myeloma [8, 9]. However, several studies suggested that JNK1 and JNK2 might also inhibit tumor progression. For example, JNK1-deficient mice are highly susceptible to intestinal cancer [10]. Moreover, JNK2 inhibits breast cancer progression via regulation of cell cycle and DNA repair [11]. It is thus important to define the specific roles played by JNKs in particular cancer types.

P53, a classic tumor-suppressor protein, inhibits the development of cancer by promoting apoptotic cell death or blocking cell cycle progression [12]. In addition, p53 is an important regulator of metabolic changes and homoeostasis, making it perhaps the most commonly mutated protein in cancer [13]. Given the high mutation rate of p53 and its various functions, it is still unclear whether p53 mutation may serve as a prognostic marker for bladder cancer patients [14]. Here, we report that JNK2 mRNA is downregulated in an orthotopic rat model of bladder cancer, and that JNK2 protein levels are decreased in rat and human bladder cancer tissues, and correlate with p53 levels. Our findings indicate that JNK2 phosphorylates p53 at Thr81, which prevents its MDM2-induced proteasome degradation, and that lower expression of JNK2 is associated with worse overall survival among patients undergoing radical cystectomy.

## RESULTS

### JNK2 expression is decreased in bladder cancer

**Figure 1:**
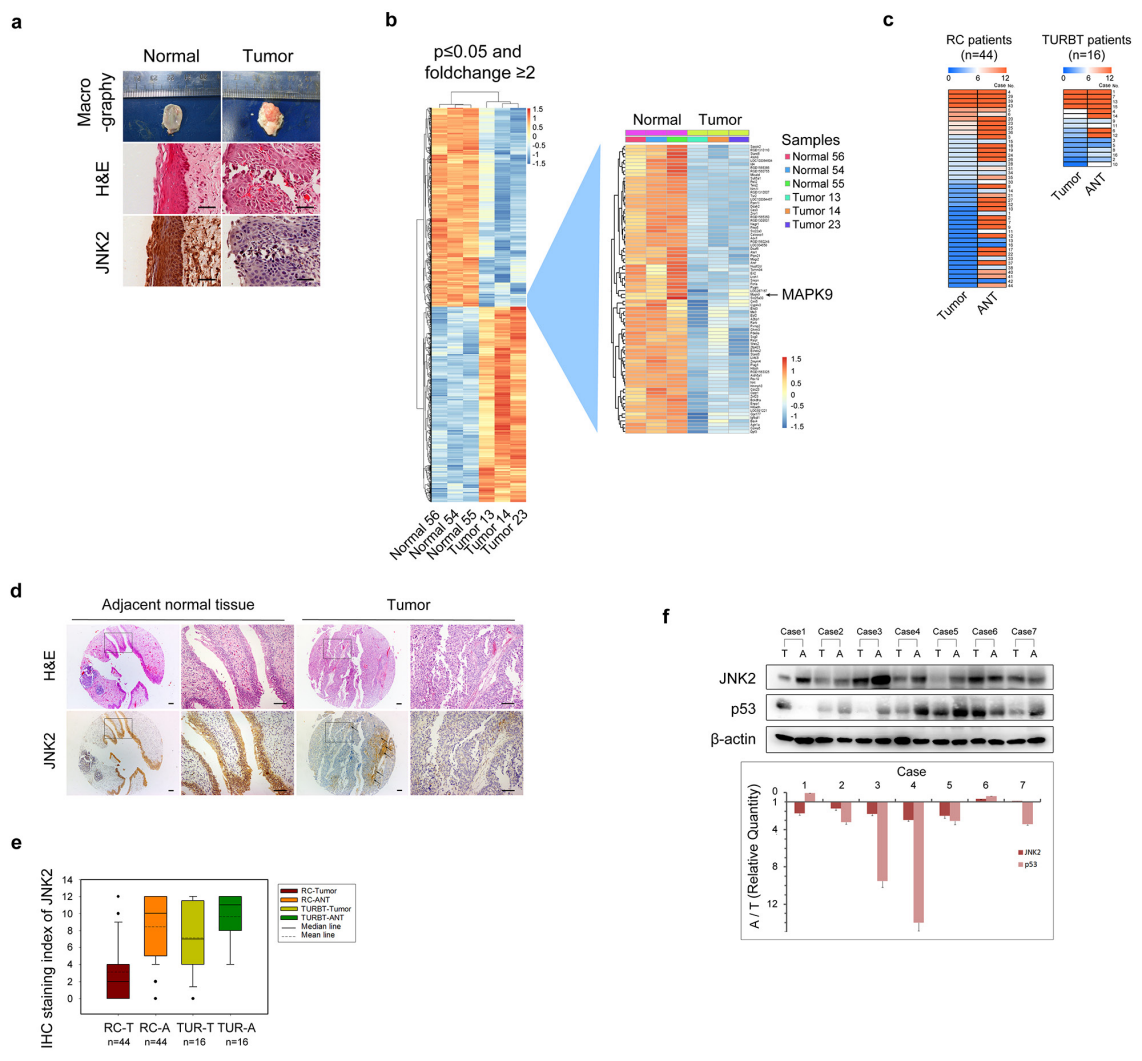
Both mRNA and protein levels of JNK2 decrease in bladder cancer **a.** Representative images of macrography, H&E staining and IHC of anti-JNK2 antibody on tissues of orthotopic rat bladder cancer model. Scale bar represents 100μm. **b.** Left panel shows changed genes in rat bladder cancer tissues (n=3) compared with normal rat bladder tissues (n=3) analyzed by gene expression microarray (GEM). Right panel shows details of *MAPK9* gene and genes around. **c.** Heat map shows IHC staining index of JNK2 of human bladder tissues. See a detailed scoring method in Material and Methods section. Left panel comes from tissue microarray (TMA) of 44 patients underwent RC (Radical cystectomy). Right panel comes from 16 patients underwent TURBT (Transurethral resection of bladder tumor). ANT: adjacent normal tissue. **d.** Representative images of H&E staining and IHC of anti-JNK2 antibody on TMA of 44 RC patients. Areas indicated by arrows are blood vessels and connective tissues, not tumor area. Scale bar represents 100μm. **e.** Box-plot shows IHC staining index of JNK2 on tissues from 44 RC patients and 16 TURBT patients respectively. **f.** Western blotting analysis of protein from bladder tissues of 7 MIBC patients. Histogram shows ratios of relative quantity of indicated proteins from ANT to tumor tissues. T: tumor tissues. A: adjacent normal tissues.

The pathologic type of the tumors was identified as bladder transitional cell carcinoma (BTCC) by H&E staining (Figure 1a). Using Affymetrix gene expression microarrays (GEM), we compared mRNA expression in tumors and normal bladder tissues. The data obtained from GEM have been deposited in the GEO database (GEO accession number GSE76702). The results of pathway analysis and Gene Ontology (GO) analysis are shown in Supplementary Figure S1 and Supplementary Figure S2. Based on the GEM data, we found that the expression patterns of genes involved in MAPK signaling exhibited prominent changes (tumor vs. normal, p=0.009, Enrichment test). For example, mRNA expression of *MAPK9*, which is the gene encoding JNK2 kinase, was significantly lower in tumors than that in normal tissues (Figure 1b). We then examined the JNK2 protein levels in rat tissues using immunohistochemistry (IHC). The JNK2 protein levels were significantly lower in tumors than in normal tissues, which was consistent with the mRNA results (Figure 1a).

In tissues obtained from RC and TURBT patients, JNK2 expression was evaluated by tissue microarrays (TMA) and IHC. As shown in Figure 1c and Figure 1d, the staining index (SI) of JNK2 in tumors was significantly lower than that in the paired ANTs of both RC patients (p=0.000, t test) and TURBT patients (p=0.005, t test). Box plot showed that there was a further decrease tendency of JNK2 levels in RC patients compared to TURBT patients (Figure 1e). Using western blotting, we further evaluated the JNK2 protein levels in tissues of 28 MIBC patients. 21 out of 28 MIBC patients exhibited lower JNK2 protein levels in tumor tissues compared with paired ANTs (p=0.000, chi-square); 7 representative cases are shown in Figure 1f. These data indicate that bladder tumorigenesis and tumor progression are associated with decreased mRNA and protein levels of JNK2.

### JNK2 promotes stability and pro-apoptotic activity of p53 through p53 phosphorylation at Thr81

**Figure 2:**
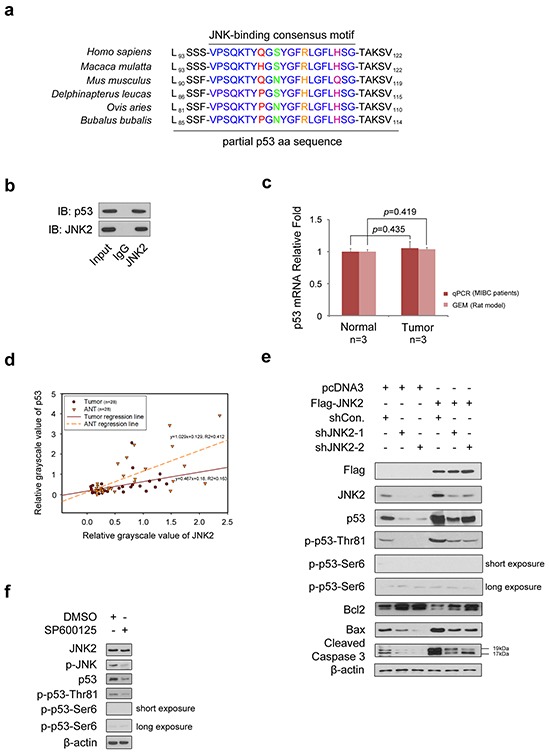
JNK2 promotes p53 stability and apoptosis activity through phosphorylation of p53 at Thr81 **a.** The p53 has consensus binding motif with JNK in different species. **b.** Western blotting detection of co-immunoprecipitated endogenous JNK2 and p53 proteins in T24 cells. **c.** Histogram shows changes of p53 mRNA between normal and tumor tissues, which are from human and rat bladder tissues respectively. **d.** Scatter plot of relative grayscale values of JNK2 and p53. Western blotting detected JNK2 and p53 proteins of tumor and paired adjacent normal tissues (ANTs) from 28 MIBC patients, then the grayscale values were measured. R^2^ in tumors and ANTs are 0.163 (p=0.033, ANOVA) and 0.412 (p=0.000, ANOVA) respectively. **e.** Western Blotting shows JNK2, p53, phosphor-p53 and related apoptotic proteins in T24 cells. Cells were infected with lentivirus expressing indicated shRNAs for 48h, then transfected with indicated plasmids for 24h before harvest. **f.** Western blot analysis of indicated proteins in T24 cells 24 h after treated with or without 10 μM SP600125.

As shown in Figure 2a, p53 primary sequence contains a JNK-binding consensus motif [5]. To determine whether p53 binds to JNK2 in T24 cells, we performed a co-immunoprecipitation (co-IP) assay (Figure 2b). We learned from the GEM data that there was no difference in *TP53* mRNA levels between normal bladder tissues and tumor tissues, which was further confirmed by real time qPCR of tissues from 3 MIBC patients (Figure 2c). However, western blotting demonstrated that the p53 protein levels were significantly lower in tumors compared to paired ANTs in 25 out of 28 MIBC patients (p=0.000,chi-square); Figure 1f shows 7 representative cases. Scatter plot of the grayscale values of JNK2 and p53 was utilized to reveal the regression relationship between p53 and JNK2 (Figure 2d). Linear regression analysis demonstrates that there is positive correlation between JNK2 and p53 in both tumors (R^2^=0.163, p=0.033, ANOVA) and normal tissues (R^2^=0.412, p=0.000, ANOVA).

Based on these results, we hypothesized that JNK2 may promote p53 stability and biological activity in bladder cancer. To test this hypothesis, we over-expressed JNK2 in T24 cells. As shown in Figure 2e, western blotting revealed that p53 and phospho-p53-Thr81 were increased in JNK2-overexpressing cells (lane 4). The Bcl-2/Bax proteins play an important role in regulating cell apoptosis, and caspase-3, a major regulator of apoptosis, is activated by cleavage of procaspase-3 during apoptosis [17]. As shown in Figure 2e, in JNK2 overexpressing cells, Bcl-2 protein levels decreased, while Bax increased. In consequence, cleaved caspase-3 was elevated (lane 4). On the other hand, when JNK2 was knocked down in T24 cells, total p53 and phospho-p53-Thr81 protein levels decreased, and the apoptosis pathway was inhibited (lanes 2 and 3). Moreover, the effect of JNK2 knockdown could be offset by overexpression of JNK2 (lanes 5 and 6). It is noteworthy that we detected only a very low signal of phospho-p53-Ser6 in these experiments. When phospho-JNK was inhibited by treating T24 cells with JNK inhibitor SP600125, total p53 and phospho-p53-Thr81 protein levels decreased, but phospho-p53-Ser6 was not detected (Figure 2f). Our findings suggest that in bladder cancer, JNK2 promotes p53 stability and downstream apoptosis signaling through phosphorylation of p53 at Thr81 site rather than at Ser6 site.

### JNK2 prevents p53 from MDM2-mediated degradation

**Figure 3:**
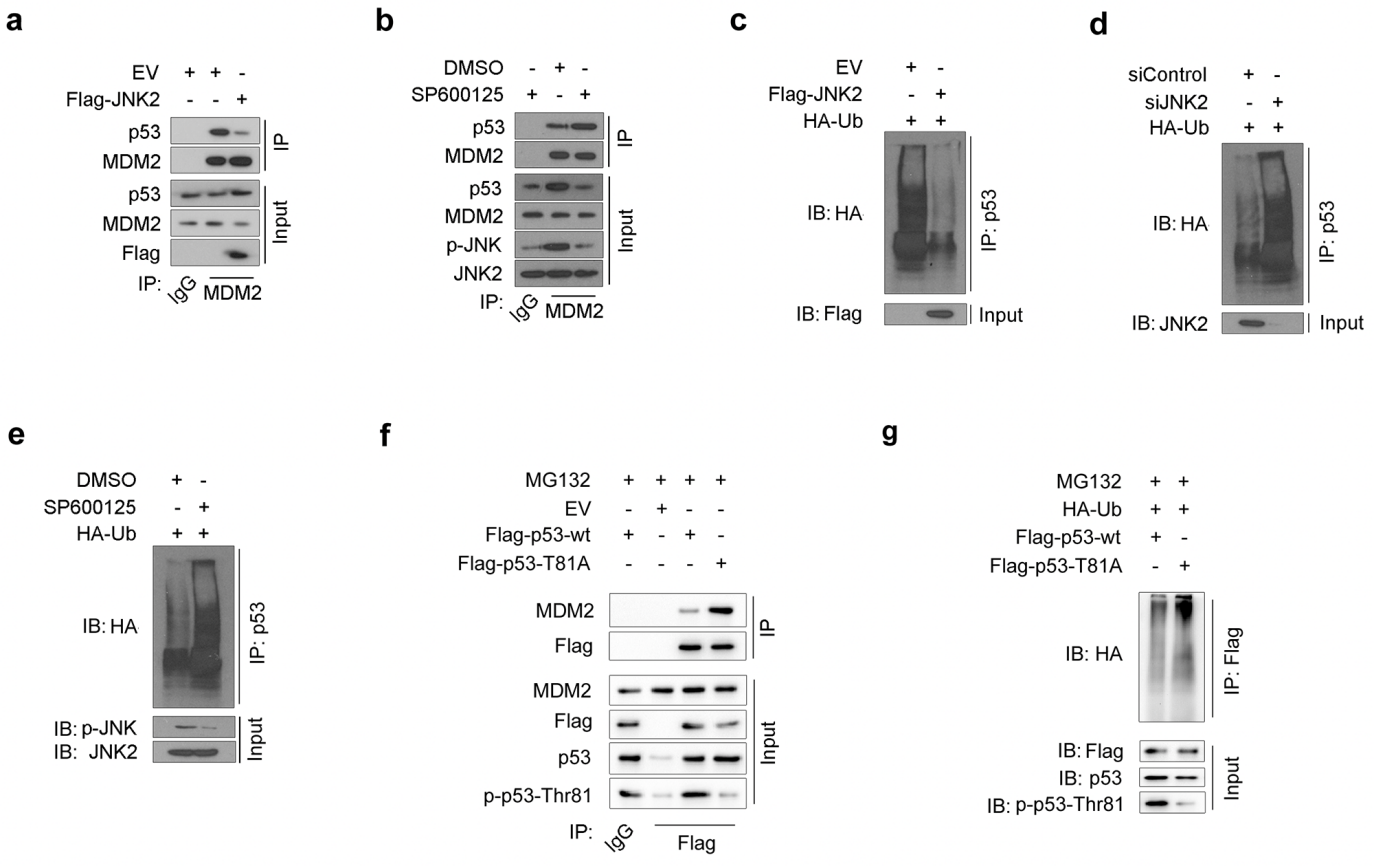
JNK2 prevents p53 from mdm2 mediated degradation **a.** Western blot analysis of whole cell lysate (WCL) and co-IP samples of IgG or anti-MDM2 antibody obtained from T24 cells 24 h after transfected with indicated plasmids. **b.** Western blot analysis of WCL and co-IP samples of IgG or anti-MDM2 antibody obtained from T24 cells 24 h after treated with or without 10 μM SP600125. **c.** Western blotting analysis of ubiquitin co-IP samples of anti-p53 antibody in T24 cells 24h after transfected with indicated plasmids. **d.** Western blotting analysis of ubiquitin co-IP samples of anti-p53 antibody in T24 cells 48h after transfected with indicated siRNAs. **e.** Western blotting analysis of ubiquitin co-IP samples of anti-p53 antibody in T24 cells 24h after treated with or without 10 μM SP600125. **f.** Western blot analysis of WCL and co-IP samples of IgG or anti-Flag antibody obtained from T24 cells 24 h after transfected with indicated plasmids. The cells were treated with 20μM MG132 for 6 hours before they were harvested. **g.** Western blotting analysis of ubiquitin co-IP samples of anti-Flag antibody in T24 cells 24h after transfected with indicated plasmids. The cells were treated with 20μM MG132 for 6 hours before they were harvested.

MDM2-mediated ubiquitination of p53 is one of the most common mechanisms of p53 inactivation. MDM2 binding to p53 and subsequent proteasome-dependent p53 degradation are important steps in inducing p53 loss in cancer cells [18]. In our study, JNK2-overexpressing T24 cells exhibited lower levels of the immunoprecipitated MDM2-p53 complex than control cells (Figure 3a). On the contrary, when T24 cells were treated with SP600125, a JNK inhibitor, p-JNK level decreased and MDM2-p53 complex increased (Figure 3b). These results indicate that p-JNK2 can prevent MDM2 from binding to p53 to form complex.

As MDM2-p53 complex is a prerequisite for p53 proteasome-dependent degradation, we measured the levels of ubiquitin binding to p53 under different JNK2 conditions. The amount of ubiquitin binding to p53 was significantly decreased in JNK2-overexpressing T24 cells (Figure 3c). Furthermore, ubiquitin binding to p53 increased when JNK2 was suppressed by siJNK2 or SP600125 (Figure 3d and Figure 3e). These results indicate that JNK2 prevents p53 from MDM2-dependent ubiquitination by blocking the MDM2-p53 complex formation.

Since our results indicate that JNK2 promotes p53 stability through phosphorylation of p53 at Thr81 (Figure 2e, 2f), and that phosphatase treatment increases the MDM2-p53 interaction (Supplementary Figure S3), we constructed a p53 Thr81Ala mutant to determine whether p53 phosphorylation at Thr81 is important for blocking the MDM2-p53 interaction. As shown in Figure 3f, p53 T81A mutant pulled more MDM2 comparing with p53 wild type (wt) control. Moreover, the amount of ubiquitin binding to p53 T81A was increased compared to p53 wt in T24 cells (Figure 3g). These results indicate that p53 phosphorylation at Thr81 is essential for blocking the p53-MDM2 interaction.

### Decreased expression of JNK2 confers resistance to cell death induced by mitomycin C

**Figure 4:**
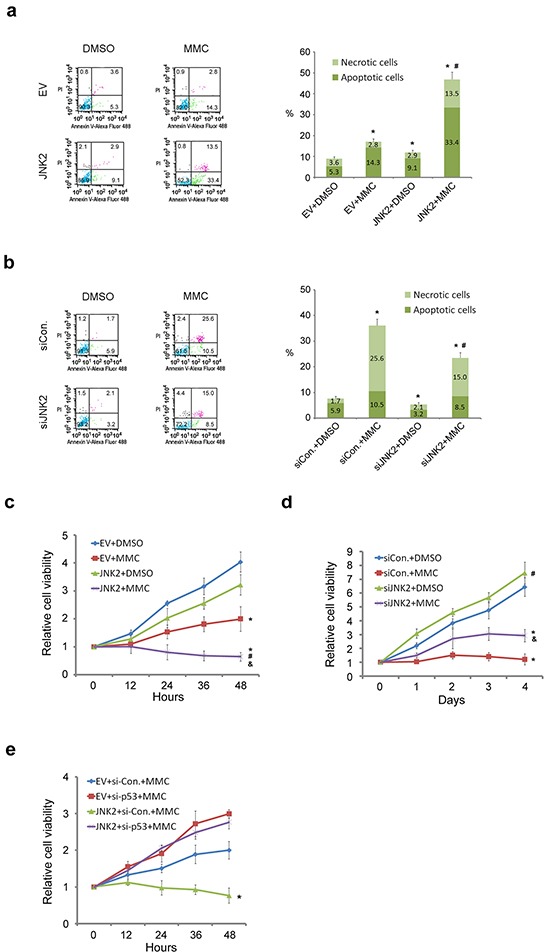
Decreased expression of JNK2 confers resistance to cell death induced by MMC (**a.** and **b**.) Flow cytometry (FCM) analysis of apoptotic and necrotic T24 cells. Cells were transfected with indicated plasmids (24h) or siRNAs (48h), then treated with or without 100ug/ml mitomycin C (MMC) for 12h before dual-stained by Annexin V-Alexa Fluor 488 and PI solution. Stained cell suspension was analyzed by FCM. *: p<0.001 vs. control group; #: p<0.001 vs. EV+MMC group. (**c** and **d**) MTS assay analysis of T24 cells proliferation. Cells were transfected with indicated plasmids (24h) or siRNAs (48h) before treated with or without 100ug/ml MMC for 12h followed by MTS assay. *: p<0.05 vs. Group 1; #: p<0.05 vs. Group 2; &: p<0.05 vs. Group 3. **e.** MTS assay analysis of 5637 cells proliferation. Cells were transfected with indicated plasmids (24h) or siRNAs (48h) before treated with 100ug/ml MMC for 12h followed by MTS assay. *: p<0.05 vs. JNK2+si-P53+MMC group.

To determine the role of JNK2 in regulating resistance to mitomycin C (MMC)-induced cell death, we measured apoptosis in T24 cells with suppressed and over-expressed JNK2. Flow cytometry analysis demonstrated that the percentage of apoptotic and necrotic cells was significantly lower in control cells than in JNK2-overexpressed cells (p=0.000, chi-square). Furthermore, the combination of overexpression of JNK2 with mitomycin C synergistically induced cell death and resulted in higher percentage of apoptotic cells than control (p=0.000, chi-square) or MMC group (p=0.000, chi-square) (Figure 4a). On the contrary, JNK2 knockdown decreased apoptosis and necrosis compared to control (p=0.000, chi-square), and siJNK2 combined with mitomycin C resulted in even lower percentage of apoptotic cells than MMC alone (p=0.000, chi-square) (Figure 4b). Consistent with these results, cell proliferation MTS assay further demonstrated that combination of mitomycin C with JNK2 overexpression resulted in greater inhibitory effect on cell viability compared to mitomycin C alone (Figure 4c). Moreover, JNK2-knockdown cells showed significantly higher viability than cells treated with mitomycin C (Figure 4d). Collectively, these data suggest that decreased expression of JNK2 confers resistance to cell death induced by mitomycin C. To determine whether the JNK2 function in regulating mitomycin C resistance is p53-dependent, we knocked down p53 and measured cell viability. MTS assay demonstrated that the synergistic effect of JNK2 and mitomycin C was reversed by p53 suppression in 5637 cells (Figure 4e). These data indicate that the resistance induced by JNK2 suppression is p53 dependent.

### Lower JNK2 expression is associated with worse clinical outcome in radical cystectomy patients

**Figure 5:**
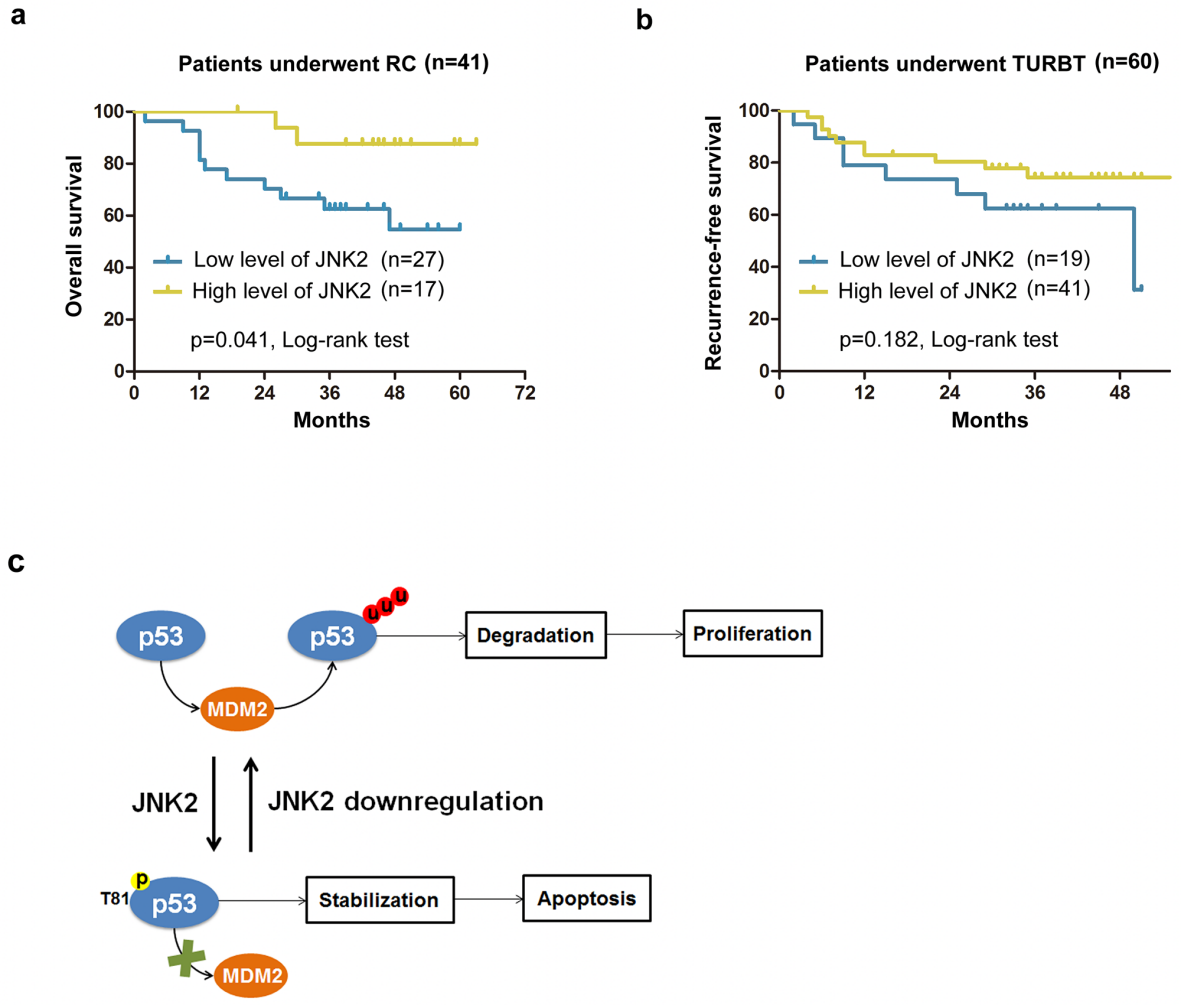
Survival analysis for patients with different expression levels of JNK2 **a.** Kaplan-Meier analysis of overall survival of patients underwent RC (Radical cystectomy) in different JNK2 conditions. **b.** Kaplan-Meier analysis of recurrence-free survival of patients underwent TURBT (Transurethral resection of bladder tumor) in different JNK2 conditions. HR=0.516 (95%CI: 0.303-0.930). **c.** A hypothetical model depicts that JNK2 serves as a tumor suppressor in bladder cancer, and that decreased JNK2 expression promotes bladder tumorigenesis.

The characteristics of patients who underwent operations are shown in Tables 1 and 2. In patients who underwent radical cystectomy (RC), the mean overall survival time of lower JNK2 expression group was 42.7±4.2months, which was markedly lower compared to the higher JNK2 expression group (58.6±2.9months) (p=0.041, Log-rank test) (Figure 5a). In patients who underwent transurethral resection of bladder tumor (TURBT), the mean recurrence-free survival time of the lower JNK2 expression group was 37.2±4.2 months, which was lower than the higher JNK2 expression group (41.7±2.6months); however, there was no statistical significance (p=0.182, Log-rank test) (Figure 5b). Cox hazard regression analysis showed that the Hazard Ratio (HR) is 0.516 (95%CI: 0.303-0.930). These data indicate that lower expression of JNK2 is associated with worse clinical outcome in RC patients.

**Table 1:** Characteristics of patients who underwent RC

Characteristics	JNK2		p value
	Low (n=27)	High (n=17)	
Age (yr)	63.6±11.1	66.8±11.0	0.303
Gender			0.359
male	25	14	
female	2	3	
Tumor grade			0.186
Low grade	2	4	
High grade	25	13	
pT stage			0.525
T1	7	4	
T2	16	13	
T3	2	0	
T4	2	0	
Lymph node status			0.455
N0	21	15	
N1, N2	6	2	
Metastasis	0	0	-

All patients who needed adjuvant chemotherapy or radiotherapy according to AUA guidelines completed these therapies.

**Table 2:** Characteristics of patients who underwent TURBT

Characteristics	JNK2		p value
	Low (n=19)	High (n=41)	
Age (yr)	72.0±10.7	67.3±14.8	0.228
Gender			
male	15	33	1.000
female	4	8	
Tumor grade			**0.013**
Low grade	6	27	
High grade	13	14	
Multifocality			0.155
Yes	13	20	
No	6	21	
Tumor size			0.194
<3 cm	17	30	
≥3cm	2	11	
pT stage			0.116
Ta	10	30	
T1	9	11	
Pedicle			**0.016**
Yes	11	36	
No	8	5	

All patients completed intravesical instillation chemotherapy according to AUA guidelines.

## DISCUSSION

Our findings suggest that JNK2 serves as a tumor suppressor in bladder cancer, and that decreased JNK2 expression promotes bladder tumorigenesis (Figure 5c). JNK2 protects p53 from MDM2 mediated degradation through phosphorylation at Thr81. Cell death induced by MMC can be attenuated by downregulation of JNK2 in T24 cells. Additionally, we found that lower expression of JNK2 is associated with poorer overall survival among patients who underwent RC.

The role of JNK2 in tumors is still controversial. Studies suggest that the actions of JNK2 is pro-tumorigenic multiple myeloma, glioblastoma, epidermal neoplasia, lung cancer, and breast cancer [9, 19–22]. However, in some lung and breast cancers, JNK2 appears to function as a tumor suppressor [23, 24]. Our study demonstrates that JNK2 also serves as tumor suppressor in bladder cancer. The GEM data from our rat bladder cancer model suggest that *MAPK9* gene is down-regulated in rat bladder tumors as compared with normal bladder tissues, which is consistent with results from other human gene expression data sets (http://www.ncbi.nlm.nih.gov/geo/, GSE13507; https://www.oncomine.org/, TCGA bladder, reporter: 05-179622439). How can JNK2 exert opposing effects, sometimes even within the same cancer type? Tumor stage-specific actions and different splice variants of JNK2 provide a partial explanation to this apparent paradox [25, 26]. JNKs are frequently activated by stress stimuli, such as cytokines, ultraviolet irradiation, heat shock, and osmotic shock [27]. Our orthotopic rat model of bladder cancer was induced by transurethral instillation of MNU, a carcinogenic stimulus, and the presence of carcinogenic chemicals in the urine is an important risk factor for human bladder cancer [28]. We therefore envision that bladder epithelial cells under stress require higher expression of JNK2 to stabilize p53, which can repair or remove impaired cells. As a result, JNK2 downregulation leads to bladder tumorigenesis.

P53 is maintained at low levels through ubiquitination and proteasomal degradation mediated mainly by the RING-finger type E3 ligase MDM2. MDM2 and p53 interact sequentially at several points of contact. A p53-binding domain in the N-terminus of MDM2 serves as a docking site for three residues in the N-terminus of p53: Phe19, Trp23 and Leu26 [29]. This high-affinity interaction leads to a conformational shift that permits the acidic domain of MDM2 to associate with the Box IV/V region of p53. This brings p53 into contact with RING domain of MDM2, leading to its ubiquitination [30]. Our findings suggest that JNK2 phosphorylation of p53 at Thr81 blocks MDM2 binding, thus reducing p53 binding to ubiquitin and, ultimately, its degradation. Buschmann et al. identified Thr81 of p53 as a JNK phosphorylation site in 293T cells [31]. However, Oleinik et al. found that p53 is directly phosphorylated by JNK2 at Ser6 in lung cancer cells [23], which is consistent with the idea that JNK2 plays different roles in different tumor types. The Thr81 phosphorylation site is not one of the N-terminus sites (Phe19, Trp23 and Leu26) or in the Box IV/V region (Ser261-Phe270) of p53. We therefore hypothesize that p53 phosphorylation at Thr81 by JNK2 produces a conformational shift that prevents p53 from binding to MDM2.

An important reason of poor clinical outcome in bladder cancer is that tumor cells become resistant to chemotherapy. In our experiments, downregulation of JNK2 inhibited p53-induced apoptotic signaling and conferred resistance to MMC-induced cell death in T24 cells. Moreover, in RC patients, overall survival time in the lower JNK2 group was significantly worse than that in the higher JNK2 group (Figure 5a). In TURBT patients, recurrence-free survival time in the lower JNK2 group also tended to be lower than in the higher JNK2 group, though the difference was not statistically significant (Figure 5b). Cox hazard regression analysis showed the HR to be 0.516 (95%CI: 0.303-0.930), implying a lower recurrence hazard in the higher JNK2 group. We should also note that in this set of TURBT patients, the overall JNK2 level was relatively high (Figure 1e), which may lead to an indistinguishable difference in recurrence rates in small samples. To show the outstanding impact on clinical outcomes of decreasing JNK2, more cases and longer follow-up will be needed in our future studies.

In sum, our data demonstrate that JNK2 is a tumor suppressor in bladder cancer, and that JNK2 downregulation promotes bladder tumorigenesis (Figure 5c). JNK2 protects p53 from MDM2-mediated degradation through phosphorylation at Thr81. Downregulation of JNK2 confers resistance to cell death induced by MMC in vitro, and lower expression of JNK2 is associated with poorer overall survival among patients who underwent RC. Collectively, our results suggest the JNK2 pathway could serve as an attractive target for therapeutic management of bladder cancer.

## MATERIALS AND METHODS

### Plasmids and reagents

The mammalian expression vectors for Flag-JNK2, Flag-p53-wt and HA-Ubiquitin were purchased from Addgene. Flag-p53-T81A was generated by site-specific mutagenesis (Stratagene). Antibodies used were anti-JNK2 (Abcam), anti-phospho-JNK (Abcam), anti-p53 (Abcam), anti-phospho-p53-Thr81 (Cell Signaling Technology), anti-phospho-p53-Ser6 (Cell Signaling Technology), anti-MDM2 (Santa Cruz Biotechnology), anti-Bcl2 (Cell Signaling Technology), anti-Bcl2 (Cell Signaling Technology), anti-Cleaved Caspase-3 (Cell Signaling Technology), anti-β-actin (Sigma-Aldrich), anti-Flag (Sigma-Aldrich) and anti-HA (Covance). SP600125 and MG132 were purchased from Selleck Chemicals, and mitomycin C was purchased from Calbiochem.

### Cell lines, cell culture, and transfection

T24 and 5637 cells were purchased from the Shanghai Cellular Institute, Chinese Academy of Science, and incubated in RPMI-1640 (HyClone) medium containing 10% fetal bovine serum (FBS) (Gibco) in a humidified chamber with 5% CO_2_ at 37°C. Transfections were performed by using Lipofectamine2000 (Invitrogen). Approximately 75–90% transfection efficiencies were routinely achieved.

### RNA interference

Non-specific control siRNA and siRNA for human JNK2 were purchased from Santa Cruz Biotechnology. Cell transfection was performed following the manufacturer's instruction. Lentivirus-based control and gene-specific shRNAs were purchased from Sigma-Aldrich (St. Lois, MO).

### Immunoprecipitation (IP) and western blotting

For IP, cells were harvested and lysed in cell lysis buffer (50 mM Tris-HCl, pH 7.5, 150 mM NaCl, 1% Nonidet P-40, 0.5% sodium deoxycholate and 1% protease inhibitor cocktails, Sigma-Aldrich) using glass dounce homogenizer. Cell lysates were centrifuged and the supernatant was incubated with indicated antibodies and protein-G beads (Invitrogen) at 4°C overnight. The beads were washed five times with cell lysis buffer and the precipitated proteins were subjected to further analysis. For western blotting, protein samples were prepared in modified RIPA buffer (1×PBS, 1% NP-40, 0.1% SDS and 1% protease inhibitor cocktail). Equal amounts of protein (50 ~ 100 μg) from cell lysates were denatured in sample buffer (Invitrogen), subjected to SDS-polyacrylamide gel electrophoresis, and transferred to nitrocellulose membranes (Bio-Rad). The membranes were immunoblotted with specific primary antibodies, horseradish peroxidase-conjugated secondary antibodies, and visualized by Super Signal West Pico Stable Peroxide Solution (Thermo Scientific).

### Ubiquitination assays

HA-ubiquitin was co-transfected with Flag-JNK2 or siJNK2 in T24 cells. The cells were treated with 20 μM MG132 (Selleck) for 6 hours before they were harvested. Cell extracts were immunoprecipitated with anti-p53 antibody. The HA-ubiquitin-p53 complex was detected by immunoblotting with the anti-HA antibody.

### Cell proliferation assay (MTS)

Cell growth was monitored by absorbance using the MTS assay according to manufacturer's instructions (Promega). Briefly, cells were plated in 96-well plates at a density of 1,000 cells per well. At the indicated times, 20 μl of CellTiter 96AQueous One Solution Reagent (Promega) was added to cells, and after 60-min incubation at 37°C, cell growth was measured in a microplate reader at 490 nm.

### Apoptosis analysis

A Vybrant Apoptosis Kit (Invitrogen) was used to measure cell apoptosis as described previously [15]. Briefly, 5 μL of Alexa Fluor 488 Annexin V and 1 μL of PI solution (100μg/mL) were added to 100 μL of cell suspension (10^6^cells/mL) and incubated for 15 min at room temperature. The stained cell suspension was analyzed by flow cytometry using FACSCalibur System (BD).

### Orthotopic rat model of bladder cancer

An orthotopic rat model of bladder cancer was established as described previously [16]. Briefly, SD rats were intravesically instilled with 20 mg/ml MNU once per week for 10 weeks. The tumor growth in bladders was monitored by CT scanning, and the pathologic type was confirmed as bladder transitional cell carcinoma (BTCC) by H&E staining.

### Gene expression microarray (GEM)

Total RNA was extracted using TRIZOL Reagent (Life technologies, Carlsbad, CA) from 3 normal and 3 tumor tissues from orthotopic rat bladder cancer model. RNA integrity was checked by an Agilent Bioanalyzer 2100 (Agilent technologies, Santa Clara, CA). Total RNA was amplified, labeled and purified by using GeneChip 3′IVT Express Kit (Affymetrix, Santa Clara, CA) to obtain biotin-labeled cRNA. Array hybridization and washings were performed using GeneChip Hybridization, Wash and Stain Kit (Affymetrix, Santa Clara, CA) in Hybridization Oven 645 (Affymetrix, Santa Clara, CA) and Fluidics Station 450 (Affymetrix, Santa Clara, CA). Slides were scanned by GeneChip Scanner 3000 (Affymetrix, Santa Clara, CA) and Command Console Software 3.1 (Affymetrix, Santa Clara, CA) with default settings. Raw data were normalized by MAS 5.0 algorithm, Gene Spring Software 11.0 (Agilent technologies, Santa Clara, CA). Data obtained from these gene expression studies are deposited in the Gene Expression Omnibus (GEO) database (http://www.ncbi.nlm.nih.gov/geo/) with the GEO accession number GSE76702.

### Real time quantitative RT-PCR (qRT-PCR)

Total RNA was isolated from tissues and cDNA was synthesized using the Super-Script kit from Invitrogen. Two-step real-time polymerase chain reaction (PCR) was performed using the SYBR Green Mix (BioRad) and iCycleriQTM detection system (Bio-Rad). Forward and reverse primers were used at a final concentration of 200 nM. The expression of *GAPDH* gene in each sample was used as an internal control. The primers for p53 were: Forward, 5′-ATG GAG GAG CCG CAG TCA GAT-3′; reverse, 5′-GCA GCG CCT CAC AAC CTC CGT C-3′.

### Human bladder tissue specimen and tissue microarray (TMA)

Forty-four BTCC patients who underwent radical cystectomy (RC) and 60 patients who underwent TURBT in Xinhua Hospital, Shanghai Jiao Tong University School of Medicine from January 2010 to December 2012 were enrolled in study. Patients with other malignant tumors or TURBT patients without following intravesical chemotherapy were excluded from the study. The tumor tissues and paired adjacent normal tissues (ANTs, 2 cm from tumor) from total of 44 RC patients were analyzed by tissue microarray (TMA) by Outdo biotechnology, Shanghai, China.

In addition, tumor tissues and paired ANTs were obtained from 28 patients who underwent RC from November 2014 to September 2015 in the same hospital. All 28 patients were diagnosed as MIBC by pathology. The tissues were stored in−80°C for later analyses by western blotting and real time qRT-PCR.

The protocols were approved by the Ethics Committee of Xinhua Hospital, Shanghai Jiao Tong University School of Medicine.

### Immunohistochemistry (IHC) and staining scoring

Tissue slides were deparaffinized through xylene and hydrated by graded alcohols after incubation at 60°C for 1 hour. Antigen retrieval by heat mediation was performed with Antigen Unmasking Solution (Vector Labs). The slides were incubated with JNK2 rabbit monoclonal antibody diluted 1:200 (Abcam) at 4°C overnight, followed by incubation with a peroxidase-conjugated goat anti-rabbit IgG (Alexa Fluor, Life technologies). The slides were visualized with Vectastain ABC kit (Vector Labs) and counterstained with hematoxylin. Control sections were incubated without the primary antibody. Staining intensity was graded/scored in a blinded fashion: 0 = no staining at 100× magnification; 1 = weak staining at 100× magnification, but little or no staining at 40×magnification; 2 = medium staining at 40×magnification; 3 = strong staining at 40×magnification. Staining percentage was also graded/scored in a blinded fashion: 1 = positive area less than 25% of the whole tissue; 2 = positive area less than 50% of the whole tissue but more than 25%; 3 = positive area less than 75% of the whole tissue but more than 50%; 4 = positive area more than 75% of the whole tissue. Staining index (SI) was obtained by multiplying values of staining percentage and intensity.

### Patients and survival analysis

Forty-four RC patients and 60 TURBT patients mentioned above were followed up from January 2010 to April 2015. Overall survival times and recurrence-free survival times were obtained. The patients were divided into 2 groups according to SI of JNK2 IHC. The SI of lower JNK2 expression group is from 0 to 5, and higher group is from 6 to 12. Log-rank test was applied and 95% confidence intervals were determined. Data were analyzed using GraphPad Prism 5.

### Statistical analysis

All experiments were done in triplicates. The chi-square test was used to compare the rates among groups. Descriptive results of continuous variables were expressed as mean ± standard deviation and assessed by Student's t test or 1-way ANOVA. The Kaplan-Meier method was used to calculate time dependent outcomes and differences were reassessed with the log rank statistic. Statistical significance was set as p<0.05. Analyses were performed with SPSS19.0.

## SUPPLEMENTARY FIGURES


