# Supplementary material for: JNK2 downregulation promotes tumorigenesis and chemoresistance by decreasing p53 stability in bladder cancer
Source: Oncotarget. 2016 Apr 27;7(23):35119–31. doi: 10.18632/oncotarget.9046 (PMC5085214; doi:10.18632/oncotarget.9046)
Supplement: Supplementary file 1 [file oncotarget-07-35119-s001.pdf]

SUPPLEMENTARY FIGURES

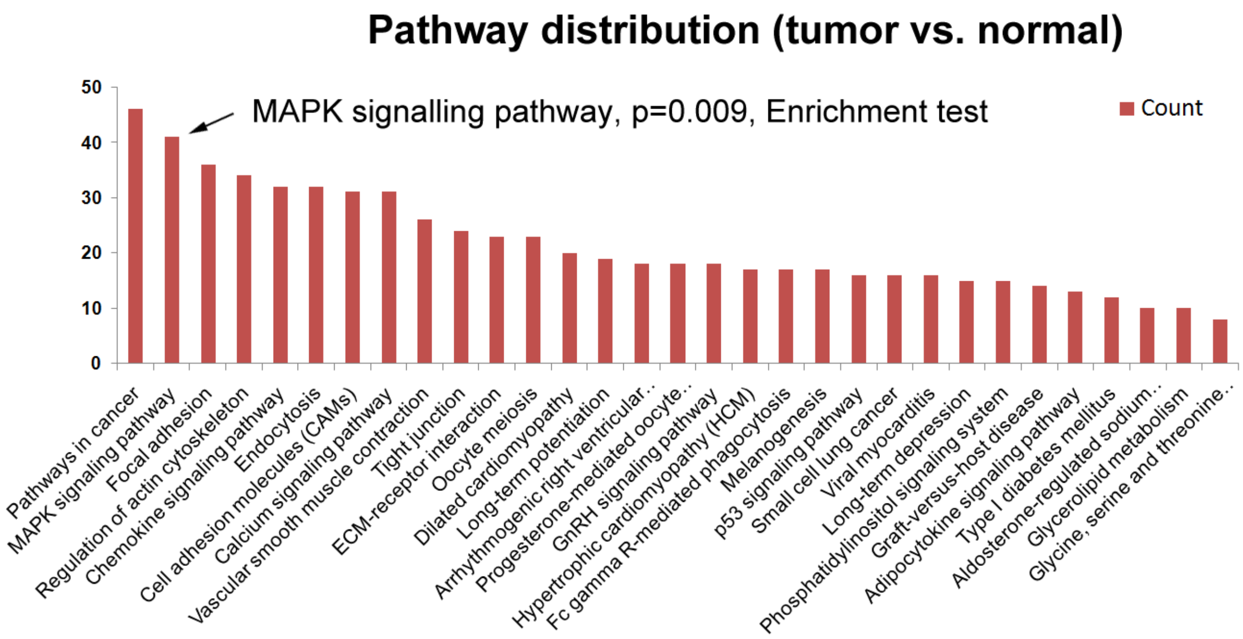

Supplementary Figure S1: Pathway analysis. From: Gene expression microarray of orthotopic rat model of bladder cancer The significant Pathway category for changed genes between the tumor and normal groups. The horizontal axis is the pathway names, and the vertical axis is the enrichment of pathways. P value<0.05 was used as a threshold to select significant pathway categories.

a

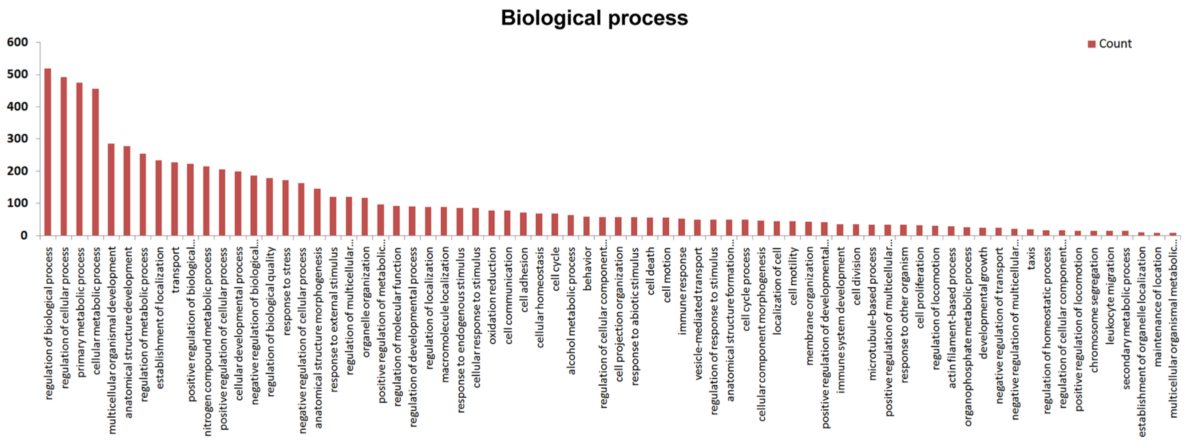

b

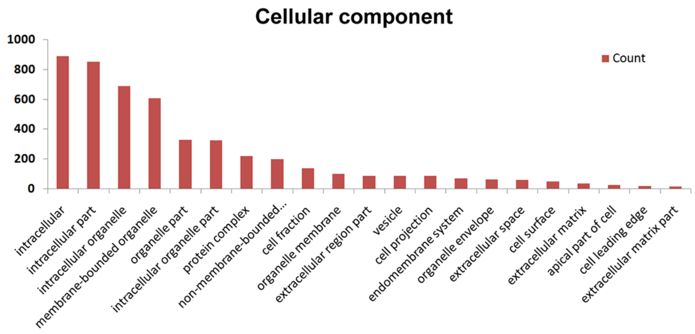

c

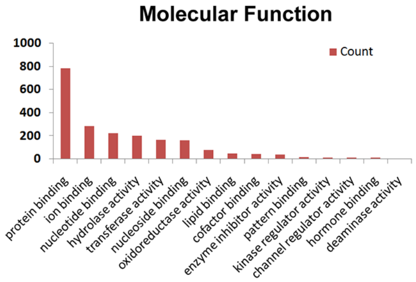

Supplementary Figure S2: Gene ontology (GO) analysis. From: Gene expression microarray of orthotopic rat model of bladder cancer The significant GO category for changed genes between the tumor and normal groups. The horizontal axis is the GO terms, and the vertical axis is the enrichment of GO. P value<0.05 was used as a threshold to select significant GO categories. **a.** GO analysis of mRNAs according to biological process. **b.** GO analysis of mRNAs according to cellular component. **c.** GO analysis of mRNAs according to molecular function.

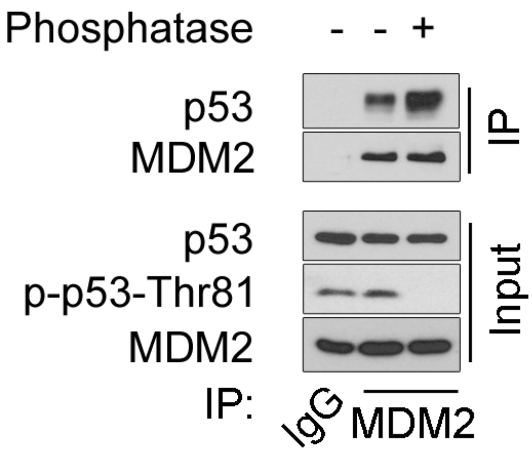

Supplementary Figure S3: Western blot analysis of T24 WCL and co-IP samples of IgG or anti-MDM2 antibody. Cell lysates were treated with or without λ phosphatase prior to IP.
